# Interplay Between the Salience and the Default Mode Network in a Social-Cognitive Task Toward a Close Other

**DOI:** 10.3389/fpsyt.2021.718400

**Published:** 2022-02-07

**Authors:** Cátia Ribeiro da Costa, Jose M. Soares, Patrícia Oliveira-Silva, Adriana Sampaio, Joana F. Coutinho

**Affiliations:** ^1^Psychological Neuroscience Lab, CIPsi – Psychology Research Center, School of Psychology, University of Minho, Braga, Portugal; ^2^Life and Health Sciences Research Institute, School of Medicine, University of Minho, Braga, Portugal; ^3^Universidade Católica Portuguesa, Human Neurobehavioral Laboratory (CEDH), Porto, Portugal

**Keywords:** social cognition, resting state, self/other processing, functional connectivity, default mode network, salience network

## Abstract

Social cognition relies on two main subsystems to construct the understanding of others, which are sustained by different social brain networks. One of these social networks is the default mode network (DMN) associated with the socio-cognitive subsystem (i.e., mentalizing), and the other is the salience network (SN) associated with the socio-affective route (i.e., empathy). The DMN and the SN are well-known resting state networks that seem to constitute a baseline for the performance of social tasks. We aimed to investigate both networks' functional connectivity (FC) pattern in the transition from resting state to social task performance. A sample of 38 participants involved in a monogamous romantic relationship completed a questionnaire of dyadic empathy and underwent an fMRI protocol that included a resting state acquisition followed by a task in which subjects watched emotional videos of their romantic partner and elaborated on their partner's (Other condition) or on their own experience (Self condition). Independent component and ROI-to-ROI correlation analysis were used to assess alterations in task-independent (Rest condition) and task-dependent (Self and Other conditions) FC. We found that the spatial FC maps of the DMN and SN evidenced the traditional regions associated with these networks in the three conditions. Anterior and posterior DMN regions exhibited increased FC during the social task performance compared to resting state. The Other condition revealed a more limited SN's connectivity in comparison to the Self and Rest conditions. The results revealed an interplay between the main nodes of the DMN and the core regions of the SN, particularly evident in the Self and Other conditions.

## Introduction

Humans are highly social beings whose general welfare depends on the quality of the relationships established with others. Social cognition (SC) is thus a fundamental ability underlying the most significant human interactions, allowing us to understand our own and others' mental states, anticipate their actions, and act accordingly (1–3). This ability is essential for adaptive interpersonal relationships, including those that we establish with significant close others. Therefore, SC is also crucial for the context of romantic relationships, whose healthy functioning critically depends on the partners' social-cognitive skills. For instance, partners who try to understand, share, and respond to the other's feelings tend to be more satisfied with the relationship (4–6).

In the field of social neuroscience, SC is conceptualized as a multidimensional construct that relies on two main subsystems, or routes, to construct the understanding of others with whom we interact (7, 8). The affective subsystem, commonly referred to in the literature as empathy or affective empathy, is responsible for our ability to experience or share the other person's emotional states (7, 9, 10). The cognitive subsystem, generally addressed as mentalizing, theory of mind, or cognitive empathy, is responsible for our capacity to cognitively represent and understand others' mental and affective states (10–12). Thus, SC involves both low-level embodied processes and high-level inference-based processes.

Over the last decades, researchers have relayed on imaging techniques, such as functional magnetic resonance imaging (fMRI), to investigate the neural basis of these two routes of social processing. Several experimental studies using different social tasks (7, 13, 14), and recent metanalysis (2, 15), have shown that empathy and mentalizing are subserved by different functional brain networks, which have also been replicated in resting state studies (7, 15). Furthermore, a study by Valk et al. (16) revealed that this dissociation between the affective and cognitive subsystems can also be observed at the brain structural level.

The affective subsystem of SC has been mainly associated with regions such as the anterior insula (AI), inferior frontal gyrus (IFG), anterior (ACC) and middle cingulate cortex (MCC), supplementary motor area (SMA), amygdala, and thalamus (7, 17–20). These regions largely overlap with the salience network (SN), a resting state network anchored in the AI and dorsal ACC (dACC) that also comprises the amygdala, ventral striatum, and the substantia nigra/ventral tegmental area (21–23).

The SN is responsible for salience attribution and integration of internal (autonomic, visceral, and somatic) and external cues to guide the emotional, interpersonal, and self-processing (22, 24, 25). The AI and dACC are typically associated with socio-affective tasks involving general forms of empathy, empathy for pain, and other interoceptive processes (26–28). For example, a study by Cheng et al. (13) demonstrated that both regions were highly activated when the participants had to imagine a loved one in pain, compared to imagining a stranger in the same situation, which was replicated in a more recent work by López-Solà et al. (29).

As pointed by Nomi et al. (30), the AI is a specific hub for affective processing and cognitive control, with functional connections to frontal, anterior cingulate, and parietal regions. Furthermore, coactivations of both the AI and ACC are observed during the emotional processing of a wide range of states from disgust to fear or anger (31), which highlights the role of the SN in the affective subsystem of SC.

On the other hand, the cognitive subsystem is subserved by a series of brain regions associated with the mental representations of ourselves and others, namely the medial prefrontal cortex (MPFC), posterior cingulate cortex (PCC) and adjacent precuneus, temporoparietal junction (TPJ), temporal pole (TP), superior temporal sulcus (STS), and inferior parietal lobule (IPL) (7, 18, 32). These regions present a clear anatomical overlap with the brain's default mode network (DMN), one of the most studied resting state networks, that normally exhibits higher activity at rest than during task performance (32–36). Notably, some psychological tasks yield little or no deactivation of the DMN when compared to resting periods (37), being that the DMN remains consistently activated in a wide range of socio-cognitive tasks such as mentalizing and mental state attribution, emotion processing, moral cognition, and episodic and autobiographic memory, among others (18, 21, 38–40).

In fact, the connection between the DMN and SC was consistently reported in various studies (41), including our own, in which we showed its positive association with pro-social personality traits like extraversion and agreeableness, both at the functional (42) and structural level (43), as well as with self-perceived empathy (44, 45). Taken together, these findings support the key role of this network for our ability to infer emotional and cognitive states.

The close relationship between resting state networks and SC, especially with the DMN, has led some authors to suggest that the brain's dynamics at rest may work as a physiological baseline that prepares us to adaptively respond to things social in nature, the most behaviorally relevant stimuli for humans (46–48). This is in line with data showing that the resting state activity facilitates subsequent social task performance activity (49).

In sum, evidence from both task performance and resting state highlights the role of the two SC-related resting state networks to construct the understanding of ourselves and others. What is less known, however, is how the functional organization of these social brain networks changes in the transition from rest to the performance of a social task, either in terms of the reconfiguration of each network's architecture and in terms of the dynamic interactions between both networks. Thus, the present study was designed to address this question by looking at the changes that occur in the transition from resting state to task performance within each network (changes in the connectivity between its nodes), as well as the changes in the interplay between the DMN—as a top-down mentalizing brain network—and the SN—as a bottom-up affective processing network. Importantly, the social task under study includes a self and close other (intimate partner) condition. The great emotional proximity with the target should influence the configuration of the networks under study due to the known anatomical overlap between self and close other processing (50). For example, the MPFC, a DMN region known to be particularly active when thinking about the self (51), is also active when thinking about a close other, particularly the ventral portion (52). Likewise, Courtney and Meyer (53), in their work about how the brain organizes representations of others based on their proximity to the self, reported a self-other overlap in the main DMN's nodes, such as MPFC and PCC/precuneus.

In terms of the interplay between the DMN and the SN, once most real social situations require both emotional sharing and mental state understanding abilities, it should be expected a significant cross-network interaction during the performance of socio-cognitive tasks, as demonstrated by previous studies (54, 55). For example, a study by Meyer et al. (56) found significant FC between the MPFC and dACC and insula in situations where participants observed a friend experiencing social exclusion compared to a stranger. In the same line, Kanske et al. (8) demonstrated that the two networks appeared to interact during the performance of a social task. Specifically, they found that during highly emotional situations, the AI inhibited the TPJ activity—a DMN's region involved in the cognitive representation of both self and other's internal states and self-other distinction (57, 58)—which, according to the authors, may indicate that in situations where empathizing and mentalizing are required, the former ability may be prioritized over the latter.

In the present study, we used two complementary approaches to analyze the pattern of FC: independent component analysis (ICA), a purely data-driven method that provides information about whole-brain functional networks (59, 60), to analyze each network's pattern of FC across the different conditions, and ROI-to-ROI correlation analysis, a method used to characterize the connectivity between pairs of predefined regions of interest (ROIs) (61), to study the interplay between the networks. To the best of our knowledge, this is the first study to use an ROI-to-ROI approach to study the FC between the DMN and the SN across different brain states. Here, we consider the SN to be mainly composed by the AI and dACC (24) and the DMN to be mainly composed by the MPFC, PCC/precuneus, and TPJ (32, 35).

Regarding the FC of the DMN across conditions, we hypothesize that the spatial maps of the DMN extracted using ICA will present the traditional nodes composing the network in the three blocks (Rest, Self, and Other). Due to the nature of the social task, which requires a clear mentalizing content, the FC of the DMN may even increase in the transition from rest to task, that is, the mentalizing regions traditionally composing the network, namely MPFC, PCC/precuneus, and TPJ, will exhibit greater FC in the Self and Other conditions in comparison to Rest.

In what concerns the SN, we also hypothesize that we will be able to observe the typical functional connectivity map of this network during Rest, Self, and Other processing. Moreover, due to the role of the SN, namely the AI and ACC nodes, for self-interoceptive processes and for the integration of physiological changes and bodily sensations, we expect to find a greater FC in these regions in the Self condition.

Regarding the interplay between networks, we expect to observe an increased connectivity between the DMN and the SN main nodes, in the Self and Other conditions, in comparison to the Rest condition. This is based on previous evidence suggesting that large-scale brain networks increase their integration as a response to task complexity (55). Additionally, we expect an increased FC between the ventral nodes of the DMN and the areas of the SN, during the Self condition in comparison to the Other condition, based on previous evidence showing an increased interplay between ventral areas of the DMN and the SN in self related processing (8, 13, 62).

Finally, in terms of how the FC of these two social brain networks relates with self-reported scores on the affective and cognitive dimensions of SC, we anticipate that the connectivity within the DMN will be positively correlated with the scores in the cognitive dimension and that the connectivity within the SN will be associated with the scores in the affective dimension.

## Materials and Methods

### Participants

Thirty-eight (17 females) Caucasian subjects who reported to be in a committed monogamous romantic relationship for at least 1 year participated in this study. The participants were recruited through a snowball sampling method. Prior to any procedure, inclusion and exclusion criteria were assessed during a preliminary screening interview conducted over the telephone. The inclusion criteria were as follows: age between 20 and 50 years old; right-handed; no prior or concurrent diagnosis of any neurological or psychiatric disorder; not dependent on alcohol and/or drugs in the last year; and ability to attend a magnetic resonance imaging (MRI) screening session (e.g., absence of metallic implants, pregnancy, etc.). The majority of the participants had college degrees (78.95%), and their ages ranged from 23 to 39 years old (*M* = 31.08, *SD* = 4.73; for males: *M* = 31.57, *SD* = 8.32; and for females *M* = 30.47, *SD* = 8.58). The mean duration of the relationship was 7.89 years (*SD* = 3.98, range = 1–15 years). Regarding relationship status, 31.58% were married couples, 36.84% were living together, and 31.58% were dating.

### Self-Report Measures

Before the experiment, participants completed a set of self-report measures of empathy and dyadic adjustment. In this study, we focused on the Portuguese version of the *Interpersonal Reactivity Index for Couples* (IRIC) to assess socio-cognitive skills in the context of the relationship. This instrument, initially developed by Péloquin and LaFountaine (5), and adapted to Portuguese by Coutinho et al. (63), is a modified version of the *Interpersonal Reactivity Index* (IRI) (64), that assesses cognitive and emotional empathy in the context of intimate relationships. It contains 13 items evaluated on a five-point Likert scale, divided into two subscales. The *dyadic perspective taking subscale* (PT) is composed of six items that measure the tendency to adopt the partner's points of view spontaneously. The *dyadic empathic concern subscale* (EC) comprises seven items and focuses on the feelings of sympathy and concerns oriented toward the partner in unfortunate situations.

The IRIC (α = 0.82) total score varies between 0 and 52, with higher scores indicating higher perceived dyadic empathy abilities. The score of PT (α = 0.85) ranges between 0 and 24, and the score of EC (α = 0.67) ranges between 0 and 28. Detailed participants' scores can be found in Table 1.

**Table 1:** Participants' IRIC total scores and respective subscales scores.

**Scale and subscales**	**Range**	***M***	***SD***
IRIC Total	32–49	40.26	4.58
IRIC-PT	7–24	16.21	3.54
IRIC-EC	19–27	24.05	2.55

*IRIC, Interpersonal Reactivity Index for Couples; PT, dyadic perspective taking subscale; EC, dyadic empathic concern subscale; M, mean; SD, standard deviation*.

### Experimental Procedure

After the first screening to assess the inclusion in the study, the goals and procedures of the study were explained to the participants, who signed a written informed consent before the beginning of the experiment. This study belongs to a large research project about social cognition in the context of romantic interaction, which was approved by the Institutional Review Board of the University of Minho and complied with the principles expressed in the Declaration of Helsinki (with the amendment of Tokyo 1975, Venice 1983, Hong Kong 1989, Somerset West 1996, Edinburgh 2000).

The experiment started with each participant completing a sociodemographic form and the self-report measures. Then, after ensuring all the security measures, each participant went on an fMRI scanning session at a clinical hospital in Oporto. While being scanned, the participants performed a social task described below. The total experimental procedure time lasted 45 min.

### Image Acquisition

Structural (T1) and functional (T2^*^) images were acquired with a clinically approved 3 Tesla MRI scanner (Siemens Magnetom Skyra, Erlangen, Germany) in one imaging session per participant. Each session included one MPRAGE T1 scan (192 sagittal slices) with the following parameters: repetition time (TR) = 2,000 ms; echo time (TE) = 2.33 s; flip angle (FA) = 7°; field of view (FoV) = 256 mm; slice gap = 0 mm; pixel size = 0.8 × 0.8 mm^2^; and slice thickness = 0.8 mm and one functional blood oxygen level depend (BOLD) sensitive echo-planar imaging (EPI) sequence (375 volumes; 39 axial slices) with the subsequent imaging parameters: TR = 2,000 ms; TE = 29 ms; FA = 90°; FoV = 1,554 mm; matrix size = 64 × 64; pixel size = 3 × 3 mm^2^; and slice thickness = 3 mm. During this sequence, the synchronization between the experimental paradigm and the acquisition for each TR was ensured using the Lumina 3G Controller. Additionally, before the experimental task, a 7-min resting state functional (T2^*^) scan (210 volumes; 39 axial slices) was acquired following the same EPI parameters. During the resting state/task free acquisition, participants were instructed to keep their eyes closed, to remain awake but relaxed and motionless as possible, doing nothing in particular.

### Socio-Cognitive Task

Each participant watched a set of short videos (20 s) of his/her romantic partner expressing emotional content. While watching the video vignettes, participants were asked to either focus on their own experience (Self condition) or on their partner's experience (Other condition). These videos, containing negative and positive emotional content toward the partner (i.e., the participant), were extracted from a previously video-recorded interaction task in the lab [details regarding this interaction task can be found in Coutinho et al. (65, 66)]. In this interaction, participants shared things that they either liked (positive content) or disliked (negative content) about their partner and vice versa.

**Figure 1:**
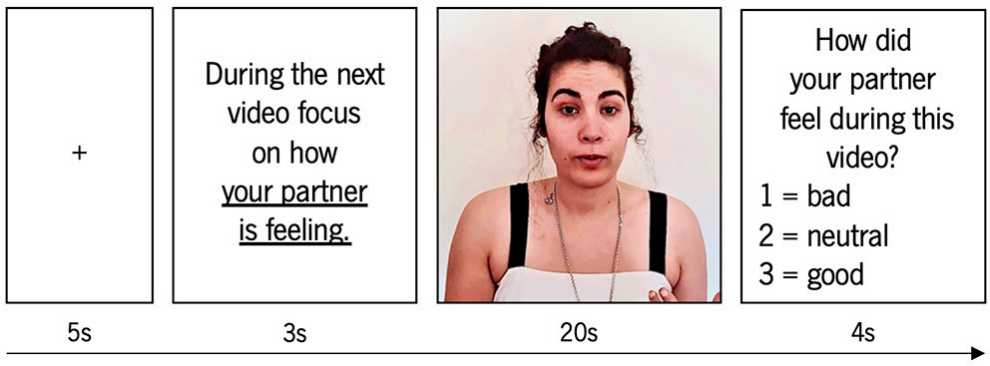
Example of a trial on the Other condition.

The task was composed of two blocks, one for each condition, and each block contained 22 trials. Each trial was composed of a fixation cross (during 5 s); instructions in accordance with each referent block (for example, the instruction for the Other block was “In the next movie focus on how your partner is feeling.”); during (3 s); video (during 20 s); and behavioral response (during 4 s). An example of a trial in the Other condition is displayed in Figure 1. The behavioral response (which aimed to ensure that participants were focusing on their own and on the partner's experience) required them to choose among one of three options, dependent on the emotional impact of the video: “Bad” for any kind of negative state or emotion, “Neutral” in the absence of any positive or negative state or emotion, or “Good” in any kind of positive state or emotion.

The stimuli were displayed in a pseudo-randomized order. The blocks were also displayed in a randomized order across participants. The total duration of the task was 1,364 s (24 min). More detailed information regarding this task can be found in Esménio et al. (50, 67).

### Data Analysis

#### Independent Component Analysis

Before data processing, all images were visually inspected to ensure the absence of head motion artifacts and any brain lesion. All imaging was preprocessed using the advanced edition of the Data Processing Assistant for Resting-State fMRI 5.1 (DPARSF; http://rfmri.org/DPARSF) (68), according to the following steps: removal of the first five volumes (10 s) to ensure signal stabilization and participant adjustment to scanner noise; slice-timing correction using the middle slice as a reference; motion correction using rigid body alignment of each volume to the mean image of the acquisition and motion scrubbing (volumes in which Frame-wise Displacement [FD] > 0.5 and DVARS > 0.5% change in the BOLD signal were “scrubbed,” or removed entirely from the data; mean group FD was 0.14 for resting, 0.15 for the Self, and 0.17 for the Other condition) to correct for movement artifacts and related susceptibility artifacts; rigid-body registration of the mean functional image to the T1 and segment using Diffeomorphic Anatomical Registration Through Exponentiated Lie Algebra (DARTEL) (69); normalization to the MNI space by DARTEL; smoothing with a Gaussian kernel of 8-mm full-width at half-maximum (FWHM) to decrease spatial noise; and band-pass temporal filtering (0.01–0.08 Hz), applied to the resting state functional images, and high-pass temporal filtering (128 s), applied to the images acquired during task performance, to remove low-frequency noise from the data.

The final images were visually inspected, and we excluded one participant due to head motion higher than 2 mm in translation and 2° in rotation for the resting state analysis, two participants due to technical problems, and one due to abnormal activation patterns/noise, for the task analysis.

Group spatial independent component analysis (ICA) was carried out to search for common spatial patterns among subjects, both during resting state and task performance, using the Group ICA v4.0c of fMRI Toolbox (GIFT; http://mialab.mrn.org/software/gift/).

The ICA consisted of extracting the individual spatial independent maps and their related time courses (70) separately for each task condition and resting state. The dimensionality reduction of the functional data and computational load was performed with principal component analysis (PCA). The estimated number of independent components (ICs) was twenty, for each subject, based on a good trade-off between preserving the information in the data while reducing its size (70, 71). ICA calculation was then performed using the iterative Infomax algorithm (72). The ICASSO tool was used to control the ICA reliability. Twenty computational runs were made on the dataset, during which the components were being recomputed and compared across runs, and the robustness of the results was ensured (73).

The ICs were obtained, and each voxel of the spatial map was expressed as a *t* statistic map, which was finally converted to a *z* statistic that characterizes the degree of correlation of the voxel signal with the component time course, providing a measure of the FC within each network. Then, the ICs were sorted, visually inspected, and spatially matched using the DMN and SN templates provided by FIND Lab (http://findlab.stanford.edu/functional_ROIs.html). We selected the IC that showed the highest spatial overlap with the provided templates to represent each network. The DMN's correlation values were 0.52 (Rest), 0.53 (Self), and 0.52 (Other), and the SN's correlation values were 0.56 (Rest), 0.26 (Self), and 0.35 (Other).

For the group analysis (second-level analysis), the general linear model (GLM) from Statistical Parametric Mapping 12.0 (SPM12; Wellcome Department of Cognitive Neurology, London, UK; http://www.fil.ion.ucl.ac.uk) was used. The individual DMN's and SN's *z* maps from each condition were included in the same group (three groups for each network, across all the conditions), and a one-sample *t-*test (*p* < 0.05 whole brain FWE corrected and extent threshold k = 10 voxels) was performed to confirm the global pattern of connectivity of the DMN and SN in the three conditions. A one-way ANOVA (*p* < 0.05 whole brain FWE corrected and extent threshold k = 10 voxels) was subsequently performed to compare the FC differences across the three conditions: Rest, Self, and Other. Subsequently, *post-hoc t*-tests were conducted to further analyze the specific differences between pairs of conditions. The resulting statistical maps were masked using the DMN and SN templates, and anatomical labeling was assigned by a combination of visual inspection and Anatomical Automatic Labeling atlas (AAL) (74).

#### ROI-to-ROI Analysis

To study the interplay between the DMN and the SN, we performed an ROI-to-ROI analysis using the CONN functional connectivity toolbox version 20.b (https://www.nitrc.org/projects/conn) (75). First, all imaging was preprocessed following the same steps described above in the ICA section. Second, we performed the ROI-to-ROI analysis (first-level analysis using GLM and applying no weight) using the DMN's and the SN' s seeds (radius of 10 mm) from the CONN database, namely the MPFC (x = 1, y = 55, z = −3), PCC/precuneus (x = 1, y = −61, z = 38), and left (x = −39, y = −77, z = 33) and right (x = 47, y = −67, z = 29) TPJ (for the DMN); and the dorsal ACC (x = 0, y = 22, z = 35) and left (x = −44, y = 13, z = 1) and right AI (x = 47, y = 14, z = 0) (for the SN) as source and target seeds. The ROI-to-ROI analysis consisted of extracting the BOLD signals from each ROI and correlated them with all the other ROIs. The correlation coefficients were converted to z-values using Fisher's transformation to improve normality. Then, a second-level analysis was performed using a one-sample *t*-test to ensure that the selected seeds were connected between each other in the three conditions, followed by a one-way ANOVA to test the FC differences between conditions. Finally, to further analyze the specific differences between conditions, we performed *post-hoc t*-tests between pairs of conditions. All results were considered significant at *p* < 0.05 whole brain FWE corrected.

#### Correlation Analysis With IRIC

The multiple regression (with positive and negative correlations) was performed, using the IC of each network at rest, to identify which areas of the DMN and the SN were associated with IRIC total, cognitive, and affective scores. Results were considered significant at *p* < 0.05 corrected for multiple comparisons using the Monte Carlo correction and a minimum cluster size of 54 for the DMN and 35 for the SN (determined over 1,000 Monte Carlo simulations using the AlphaSim program distributed with the REST software tool [http://restingfmri.sourceforge.net/] with the following input parameters: individual voxel probability threshold = 0.05, cluster connection radius = 3 mm, Gaussian filter width [FWHM] = 8 mm, and mask set to the DMN and SN templates). The resulting statistical maps were also presented using the DMN's and SN's templates as masks, and only the typical network regions were reported. Anatomical labeling was assigned by a combination of visual inspection and AAL.

## Results

### DMN's and SN's Functional Connectivity in Rest, Self, and Other Conditions

**Figure 2:**
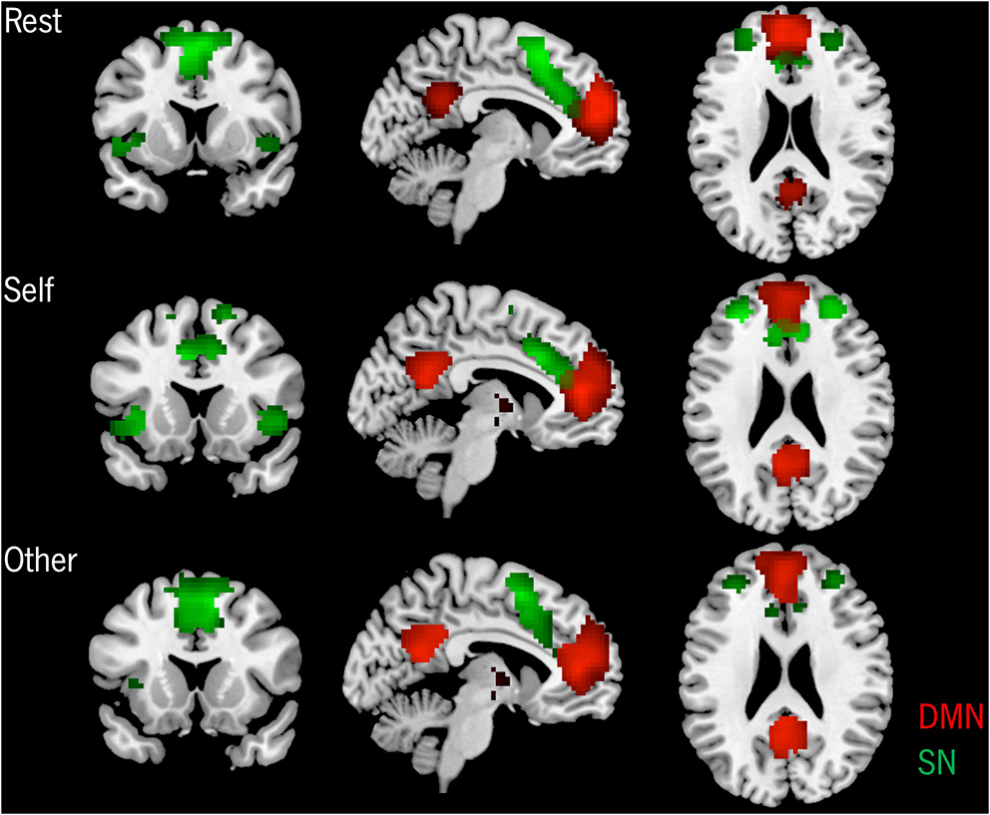
Group-level spatial patterns of the DMN and the SN in the three conditions. *p* < 0.05 FWE corrected, extent threshold *k* = 10 voxels.

At a group level, both the DMN's and the SN's spatial maps presented the traditional connectivity patterns associated with each network in the three conditions (results shown in Figure 2).

The results from the one-way ANOVA revealed a significant effect of each condition when compared to the other two, both for the DMN's and SN's functional connectivity (FC), as can be observed in Table 2. Specifically, *post-hoc t*-tests for the DMN showed an increased FC for the Self in comparison to the Rest condition on anterior and posterior regions, namely on the ventral anterior cingulate cortex (ACC), frontal medial orbital cortex (FMO), posterior cingulate cortex (PCC)/precuneus, and cuneus. An anterior and posterior DMN increased FC on the Other condition compared to the Rest was also observed on the FMO, PCC/precuneus, and left lingual/parahippocampal gyrus. On the other hand, the Rest condition only presented increased FC on anterior regions, specifically on the left superior frontal gyrus (SFG) when compared to the Self condition, and on the bilateral SFG and right superior medial frontal gyrus (SMFG) when compared to the Other condition. No significant differences were found between the DMN's connectivity on the Self and Other conditions.

**Table 2:** Condition dependent differences of the DMN and SN's functional connectivity.

		**MNI coordinates**						**MNI coordinates**						**MNI coordinates**				
	**Region of interest**	***x***	***y***	***z***	***T***	***k***	**Region of interest**	***x***	***y***	***z***	***T***	***k***	**Region of interest**	***x***	***y***	***z***	***T***	***k***
	Rest > Self and Other						Self > Rest and Other						Other > Rest and Self					
DMN	L Frontal medial orbital	0	51	−6	225.91	114	L Frontal medial orbital	0	48	−9	349.25	21	L Frontal medial orbital	0	57	0	250.58	69
	R Frontal medial orbital	9	42	−3	53.74								R Frontal medial orbital	9	42	−3	32.53	
	L Superior frontal	−12	36	51	67.61	82							L Superior medial frontal	−9	39	48	50.87	73
	R Posterior cingulate/ Precuneus	9	−66	33	55.12	32							R Superior frontal	15	39	48	42.36	12
	R Superior frontal	15	39	48	44.38	13												
	L Lingual	−15	−36	−3	40.03	27												
	L Parahippocampal	−18	−33	−12	36.64													
SN	L Supplementary motor area	−6	15	54	246.68	421	L Supplementary motor area	−6	12	54	348.68	404	L Supplementary motor area	−6	3	69	129.23	84
	R Supplementary motor area	9	6	69	69.30		R Superior frontal	21	45	21	94.55	74	R Supplementary motor area	6	3	69	101.53	
	L Anterior Insula	−45	18	−9	144.23	51	R Middle frontal	36	39	27	59.49		L Anterior insula	−39	18	−9	126.62	81
	L Middle frontal	−30	54	27	70.23	17	L Middle frontal	−36	39	27	77.74	31	R Anterior insula	39	9	−3	100.82	80
	R Anterior Insula	46	18	−6	56.65	26	R Anterior cingulate	12	36	24	51.70	23	L Anterior cingulate	−9	36	21	80.46	210
							L Anterior Insula	−36	12	−9	37.17	10	R Anterior cingulate	6	30	24	54.94	
													L Middle frontal	−24	48	27	69.35	39
													R Superior frontal	21	9	57	58.97	15
													L Superior frontal	−18	3	63	58.89	13

*p < 0.05 FWE corrected, extent threshold k = 10 voxels; MNI, Montreal Neurological Institute; k, cluster size; L, left, R, right*.

**Figure 3:**
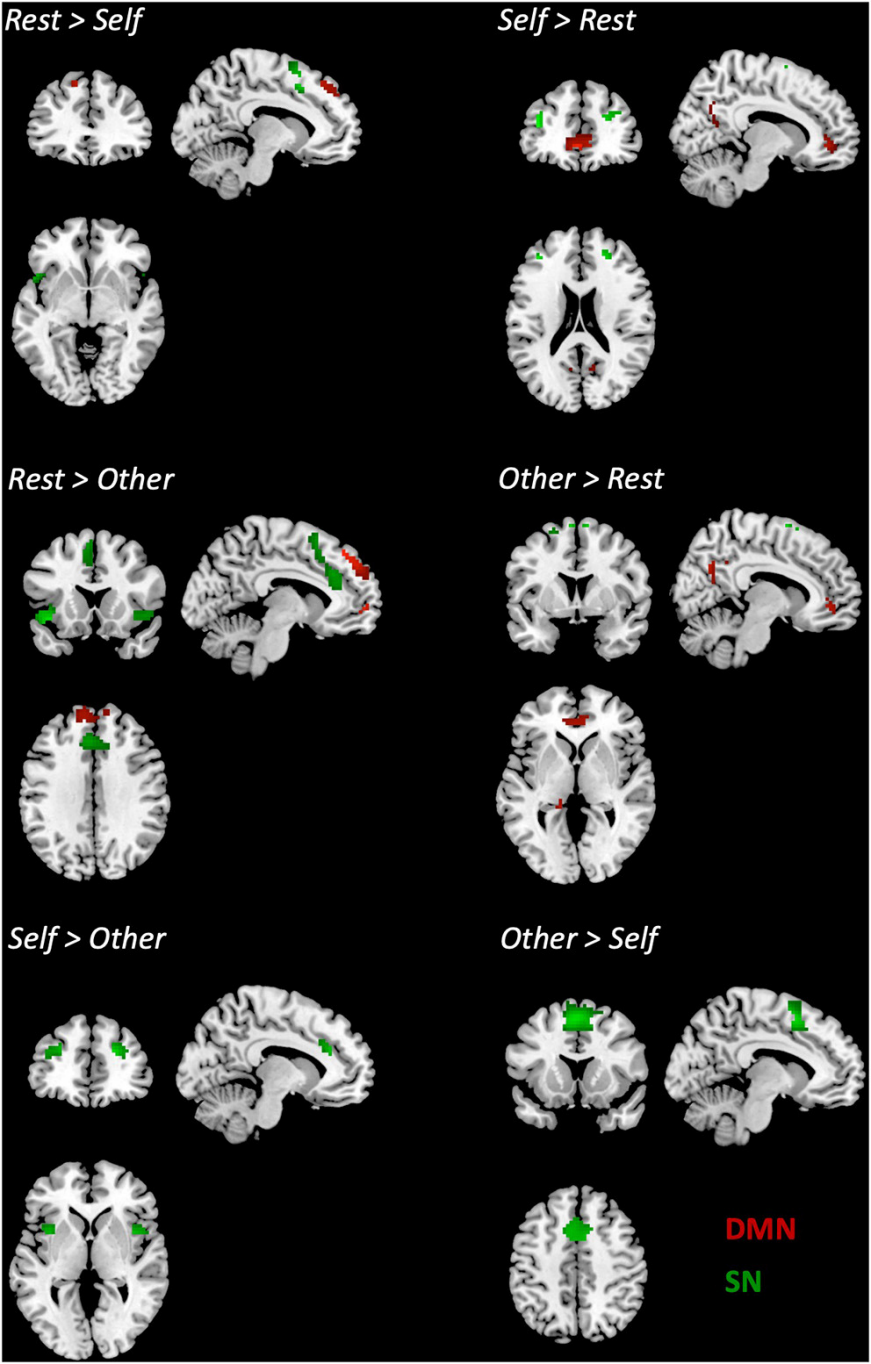
Differences in the connectivity of the DMN and the SN between pairs of conditions. *p* < 0.05 FWE corrected, extent threshold *k* = 10 voxels.

*Post-hoc t*-tests for the SN revealed an increased FC on the Self condition compared to the Rest on the bilateral middle and superior frontal regions, while on the supplementary motor area (SMA) and the superior temporal pole, the Rest condition presented higher FC compared to the Self. On the Self condition, when compared to the Other, increased FC was observed on the right SFG, anterior insula (AI), left middle frontal gyrus (MFG), and dorsal ACC (dACC). On the contrary, on the Other condition, increased FC was found on the bilateral SMA. When comparing the Rest and Other conditions, an increased FC on the AI, dACC, SMA, and on the left MFG was found on the Rest condition. On the opposite, on the Other condition, increased FC was verified on the SMA and the left SFG. Detailed results and MNI coordinates can be found in Figure 3 and Table 3.

**Table 3:** Differences in the DMN and SN'S functional connectivity between conditions.

		**MNI coordinates**						**MNI coordinates**				
	**Region of interest**	***x***	***y***	***z***	***T***	***k***	**Region of interest**	***x***	***y***	***z***	***T***	***k***
DMN	Rest > Self						Self > Rest					
	L Superior frontal	−12	36	51	6.06	32	L Ventral anterior cingulate	−3	45	−3	16.46	147
							R Frontal medial orbital	9	42	−3	8.37	
							R Posterior cingulate/Precuneus	12	−63	27	6.86	29
							L Precuneus/Cuneus	−9	−63	30	5.89	10
	Rest > Other						Other > Rest					
	L Superior frontal	−9	39	48	11.83	176	L Frontal medial orbital	0	48	−6	11.27	85
	R Superior medial frontal	3	45	45	6.80		R Frontal medial orbital	9	42	−3	6.55	
	R Superior frontal	15	39	48	7.99	17	R Posterior cingulate/Precuneus	9	−66	33	8.16	37
							L Lingual/Parahippocampal	−15	−36	−3	6.68	33
							L Posterior cingulate/Precuneus	−6	−66	33	6.17	11
SN	Rest > Self						Self > Rest					
	L Supplementary motor area	−6	15	54	18.73	416	L Middle frontal	−36	39	27	7.42	17
	L Superior temporal pole	−48	18	−12	9.56	22	L Inferior frontal triangularis	−36	42	15	6.30	
							R Superior frontal	24	42	21	6.36	33
							R Middle frontal	36	39	27	6.20	
	Self > Other						Other > Self					
	R Superior frontal	21	45	21	9.50	72	R Supplementary motor area	6	3	54	16.25	387
	L Anterior insula	−36	12	−6	8.87	55	L Supplementary motor area	−3	3	54	15.56	
	R Anterior insula	39	9	−3	8.86	51	L Middle frontal	−24	6	63	5.88	
	L Middle frontal	−27	39	24	8.22	42						
	L Dorsal anterior cingulate	−12	33	24	8.10	151						
	R Dorsal anterior cingulate	15	27	30	8.08							
	Rest > Other						Other > Rest					
	L Anterior insula	−39	18	−9	12.62	75	L Supplementary motor area	−6	3	69	12.81	15
	L Dorsal anterior cingulate	−6	30	30	8.26	245	R Supplementary motor area	6	3	69	11.35	
	L Supplementary motor area	−6	15	54	7.69		R Superior frontal	−21	6	63	6.92	10
	R Anterior insula	39	9	−3	7.98	75						
	L Middle frontal	−24	48	27	7.97	30						

*p < 0.05 FWE corrected, extent threshold k = 10 voxels; MNI, Montreal Neurological Institute; k, cluster size; L, left, R, right*.

### Interplay Between DMN and SN in Rest, Self, and Other Conditions

**Figure 4:**
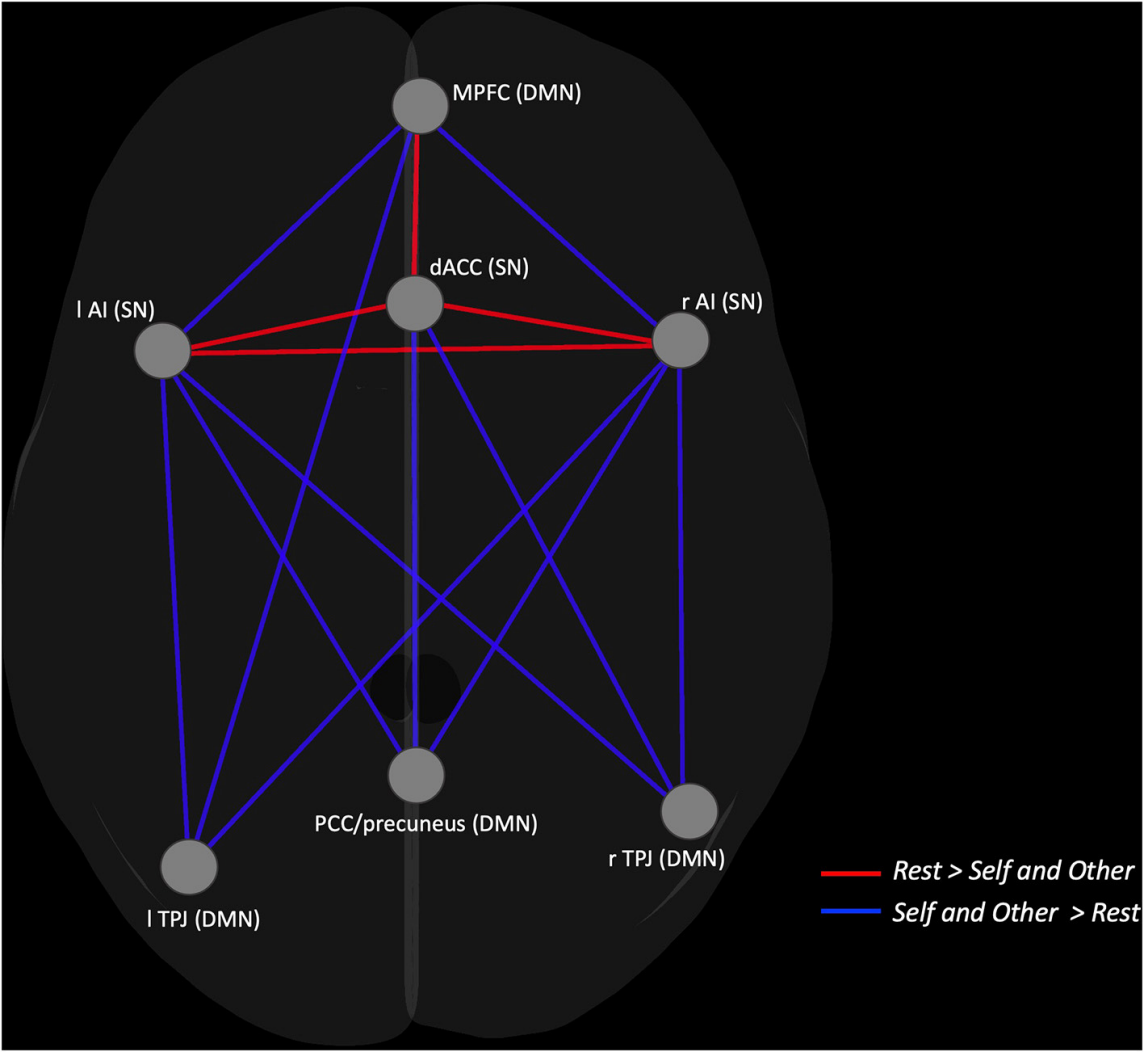
Graphical representation of the ROI-to-ROI contrast showing the nodes with increased FC in the Self and Other conditions (blue lines) compared to the Rest and increased FC in the Rest condition (red lines) compared to the Self and Other.

The results from the ROI-to-ROI correlation analysis showed an interplay between both networks in the three conditions. Furthermore, the results revealed significant increased connectivity between the ROIs of the DMN and the ROIs of the SN in both the Self and Other conditions in comparison to the Rest condition, specifically between the temporoparietal junction (TPJ) and both SN nodes, AI and dACC; between the PCC/precuneus and AI and dACC; and between the medial prefrontal cortex (MPFC) and AI. Additionally, the results show an increased intranetwork FC between the DMN nodes left TPJ and the MPFC in the task-dependent conditions when compared to the resting state. Inversely, the results revealed increased FC between the right AI and left AI, between the dACC, and AI and between the MPFC and dACC in the Rest condition, compared to the Self and Other conditions. When comparing the Self and Other conditions, no significant results were observed. Detailed results can be found in Figure 4 and Table 4.

**Table 4:** ROI-to-ROI results showing functional connectivity differences between the Rest and the Self and Other conditions.

**Seed**	**Target**	***T***	***p***	**Seed**	**Target**	***T***	***p***
Rest < Self and Other				Rest > Self and Other			
		31.12	0.001			11.66	0.038
L Temporoparietal junction	R Anterior insula	−3.86		R Anterior insula	L Anterior insula	2.75	
R Temporoparietal junction	R Anterior insula	−3.35		Dorsal Anterior cingulate	L Anterior insula	2.91	
Posterior cingulate/precuneus	R Anterior insula	−3.05		Dorsal Anterior cingulate	R Anterior insula	1.97	
Posterior cingulate /precuneus	Dorsal Anterior cingulate	−2.59		Medial prefrontal cortex	Dorsal Anterior cingulate	1.29	
R Temporoparietal junction	L Anterior insula	−2.58					
L Temporoparietal junction	L Anterior insula	−2.41					
R Temporoparietal junction	Dorsal Anterior cingulate	−1.75					
Posterior cingulate /precuneus	L Anterior insula	−1.24					
L Temporoparietal junction	MPFC	−1.38					
Medial prefrontal cortex	L Anterior insula	−1.57					
Medial prefrontal cortex	R Anterior insula	−1.53					

*p < 0.05 FWE corrected; L, left, R, right*.

### Association Between DMN's and SN's FC and Self-Report Measures

**Figure 5:**
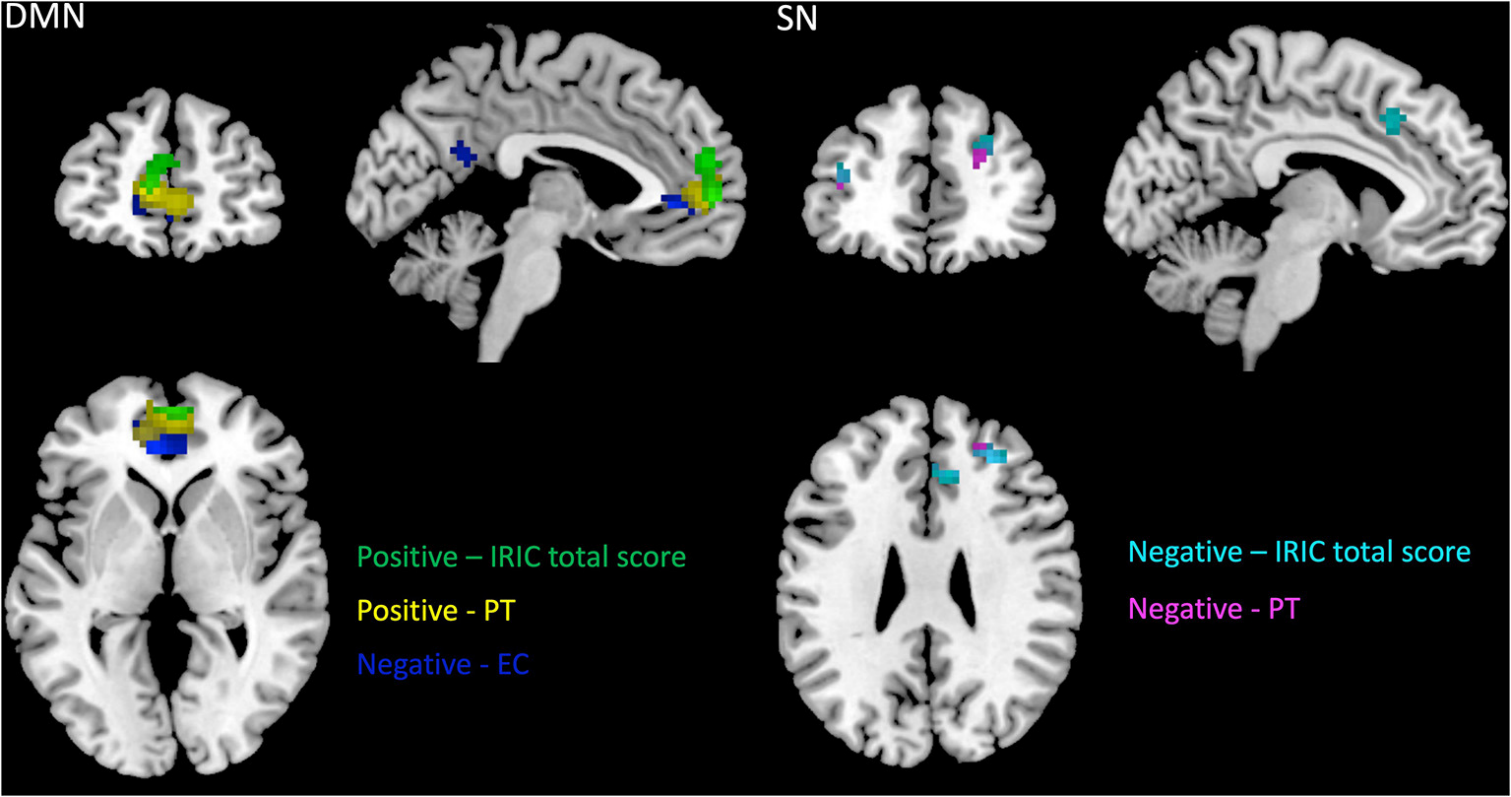
Correlations between DMN and SN functional connectivity and IRIC scores. *p* < 0.05 corrected for multiple comparisons, extent threshold of *k* = 54 voxels for DMN and *k* = 35 for SN. IRIC, Interpersonal Reactivity Index for Couples; PT, dyadic perspective taking subscale; EC,dyadic empathic concern subscale.

Regarding the correlations between the DMN's connectivity and the participant's social cognitive scores, we found that the total IRIC scores, as well as the cognitive subscale of IRIC (perspective taking), were positively correlated with the FC in the SMFG (*r* = 0.46; *r* = 0.53), whereas for the affective subscale (empathic concern), the correlation was negative, with increased FC in the ventral ACC (*r* = −0.59) and right precuneus (*r* = −0.51) being associated with lower scores in the affective domain (see Figure 5).

When considering the SN, the significant correlations with IRIC were negative, with increased FC in the right MFG (*r* = −0.50; *r* = −0.53), right dACC (*r* = −0.44), left SMFG (*r* = −0.40), and left SFG (*r* = −0.38) associated with decreased cognitive scores.

Detailed results and MNI coordinates can be seen in Figure 5 and Table 5.

**Table 5:** Correlations between DMN and SN functional connectivity and IRIC scores.

				**MNI** **coordinates**						
	**Scale**	**Correlation**	**Region of interest**	***x***	***y***	***z***	***T***	***p***	***k***	***r***
DMN	IRIC Total	Positive	R Superior medial frontal	3	60	3	3.04	0.002	146	0.46
			L Superior medial frontal	−3	54	21	2.64	0.006		0.41
	IRIC-PT	Positive	L Superior medial frontal	−12	48	6	3.69	0.000	199	0.53
	IRIC-EC	Negative	L Ventral anterior cingulate	−6	39	0	4.28	0.000	84	−0.59
			R Ventral anterior cingulate	3	42	−3	3.39	0.001		−0.50
			R Posterior cingulate/Precuneus	3	−54	24	3.55	0.001	87	−0.51
SN	IRIC Total	Negative	R Middle frontal	24	39	24	3.37	0.001	35	−0.50
	IRIC-PT	Negative	R Middle frontal	27	39	30	3.66	0.000	37	−0.53
			R Dorsal anterior cingulate	9	33	30	2.93	0.003	84	−0.44
			L Superior medial frontal	0	24	42	2.57	0.007		−0.40
			L Superior frontal	−12	18	45	2.40	0.011		−0.38

*p < 0.05 corrected for multiple comparisons, extent threshold of k = 54 voxels for DMN and k = 35 for SN; MNI, Montreal Neurological Institute; k, cluster size; L, left, R, right; IRIC, Interpersonal Reactivity Index for Couples; PT, dyadic perspective taking subscale; EC, dyadic empathic concern subscale*.

## Discussion

In the present study, we aimed to analyze the functional connectivity (FC) of the DMN and the SN, both during resting state and during the performance of a social-cognitive task toward a romantic close other. This task included a Self condition in which participants had to elaborate on their own experience and an Other condition in which they elaborated on their partner's experience. Thus, we compared the FC patterns of these two social cognition (SC) related networks in the three conditions—Rest condition, Self condition, and Other condition—using independent component analysis (ICA). In addition, we looked at the interplay between both networks across the three conditions to better understand how the dynamic interaction across the socio-cognitive (DMN) and socio-affective (SN) functional brain systems changes in the transition from rest to a social task—using an ROI-to-ROI correlational analysis.

In terms of the DMN's connectivity pattern, accessed via ICA, we found that the main nodes of the network were functionally connected in the three conditions. As expected, and considering the key role of the DMN as a mentalizing system, we found that the FC pattern changed in the transition from resting state to self and other processing, presenting increased connectivity on its anterior and posterior nodes, namely on the medial prefrontal cortex (MPFC) and posterior cingulate cortex (PCC)/precuneus during task performance in comparison to rest. These results are consistent with the metanalysis by Alcalá-López and colleagues (15) in which an increase in the strength of the DMN's intranetwork connectivity during the performance of social tasks when compared to resting state had also been reported. In the same line, a recent work by Wang and colleagues (76) on the structural and functional connectome of the social mentalizing network reported an increase in the FC of areas such as the dorsal and ventral MPFC, the temporoparietal junction (TPJ), and the precuneus when the demands of the mentalizing task increased. Furthermore, during the Other condition, increased FC on the lingual/parahippocampal gyrus was observed compared to the Rest condition. This increased connectivity in hippocampal regions during task performance may reflect the retrieval of memories of past experiences (77, 78) needed for the task in the Other condition in which subjects may have evoked specific episodic memories related to the content depicted by their romantic partner in the video vignettes.

Interestingly, the results revealed no differences in the DMN's connectivity between the Self and Other conditions, and the observed increased connectivity on the MPFC and PCC/precuneus both during the Self and Other is consistent with the results found by Courtney and Meyer (53), in which the authors reported a self-other overlap in these DMN's nodes. Overall, these findings confirm the well-known relationship between the DMN and our ability to infer internal states, either our own or those of others (41, 46, 47), in the particular case of the present study, the internal states of our romantic partner.

The SN also presented its typical pattern of FC across the three conditions. As anticipated, the observed main difference suggested a more limited FC in key nodes of the SN such as the dorsal anterior cingulate cortex (dACC) and the anterior insula (AI) on the Other condition compared to the Rest and Self conditions. Higher connectivity on these conditions suggests that either when left to think freely (Rest condition) or explicitly told to think about their internal states (Self condition), the emotional circuits subserved by the SN seem to display greater FC, which aligns with our hypothesis and the well-known association between the SN and self-referential interoceptive processes (28, 78). Moreover, a parallel for this evidence could be drawn based on the work by Cheng et al. (13), in which subjects watched painful situations and had to imagine them from a self, loved one, and stranger perspective. Although the three perspectives were related to a neural pain processing network, activation in the AI and ACC showed a gradient decline from the self to close other (to the stranger).

Having characterized and compared the functional architecture of the DMN and SN on the three different conditions, we proceeded to analyze the interplay between them. Using an ROI-to-ROI approach to see how the nodes of the DMN interact with the ones from the SN, we intended to better understand the integration between cognitive and emotional dimensions of SC during rest and during the performance of a social task. As hypothesized, our results pointed to an interplay between the two networks in the three conditions. Importantly, both self and other processing conditions showed a higher FC between the main DMN nodes—MPFC, PCC/precuneus, and TPJ—and the nodes of the SN—AI and dACC—when compared to rest, pointing to an increased functional interaction between both networks when the subjects were actively involved in the social task. This increased connectivity suggests the need for a greater integration between affective or bottom-up and cognitive or top-down dimensions during the active engagement in a social processing task. Likewise, in a review on the types of brain network organization that occurs in the context of SC, Schurz and colleagues (55) concluded that increased network integration indicated more effortful and controlled processing. Shine and collaborators (79) also found that network integration was higher in a theory of mind task (Social Animations) when compared to passive rest, leading the authors to conclude that large-scale brain networks increase their integration as a response to task complexity (80).

Previous studies have suggested that certain regions, such as the PCC (62) or the TPJ (50), tend to display increased connectivity when processing information related to the other, whereas areas such as the MPFC (49) and the AI (62) tend to show higher FC when processing self-related information. In a study of functional activation by our research team (50), in which Self and Other were also contrasted, the results revealed a self-other overlap with activations on regions such the inferior frontal and orbital gyrus, superior and inferior temporal gyrus, PCC/precuneus, fusiform gyrus, thalamus, and inferior occipital gyrus. On the other hand, the results also showed higher activations on the superior temporal gyrus and insula on the Self condition compared to the Other and, inversely, higher activations on the caudate nucleus, fusiform gyrus, middle occipital gyrus, inferior and middle temporal gyrus, supramarginal, and angular gyrus on the Other condition compared to the Self. Thus, based on these results, we expected increased FC between the selected seed regions in the Self condition in comparison with the Other condition; however, no differences were found in terms of the internetwork connectivity when comparing the conditions.

Surprisingly, we observed increased FC between the TPJ and the AI, both in the Self and Other conditions, as opposed to the Rest. The TPJ allows for rapid switching between one's own perspective and the perspective of others with whom we are relating (81), and as suggested by Qin et al. (82), connectivity between the insula and TPJ could serve the association between internal and external aspects of the self, which could serve as the basis for further co-representation of social information pertaining to both self and other. In a study where subjects observed strangers and close others experiencing a painful stimulus, Cheng et al. (13) found negative connectivity between the right TPJ and the right AI in the stranger perspective. The authors also found that the closer the relationship between the observer and the target, the greater the right TPJ deactivation and the higher the activation in the AI, which led them to conclude that the TPJ deactivation may reflect the increased self-other blending that characterizes empathic processes toward close others. The same process of inclusion of the other in the self may have influenced our results in which the target was an intimate other, leading us to infer that if we had included another experimental condition in which the target was a distant or nonfamiliar other, we would find a higher FC between the TPJ and the AI, and this difference would be more pronounced for the distant other in comparison with the Self or close Other condition.

Inversely, the SN main nodes—AI and dACC—displayed greater FC between each other in the Rest Condition, compared to the other two conditions, which highlights the role of these regions as the core nodes of the network (23, 24). This result may lead us to hypothesize that due to the unconstrained nature of the resting state instructions, subjects may have been more focused on processing their own interoceptive and somatic states, which may have required a higher integration of the two main nodes of the SN traditionally linked with interoceptive processing.

Additionally, it was only in the Rest condition that we observed an increased connectivity between the MPFC and the dACC, suggesting the existence of a stronger coupling between these two nodes of the DMN and SN at rest. This is in accordance with our hypothesis, which in turn was based on the known integration between ventral areas of the DMN and the SN during several psychological processes that may be present at rest, such as self-referential and interoceptive processing as mentioned above (13, 62). It is also in accordance with the putative modulatory role for the SN in regulating the DMN activation (21).

Finally, the greater involvement of the DMN in the cognitive route of social processing and of the SN in the affective route was confirmed by the results of the correlational analysis between their FC patterns at rest and self-perceived empathic abilities, in that the DMN was positively associated with IRIC cognitive scores and negatively associated with affective scores, whereas the SN was negatively associated with cognitive IRIC scores. This adds to a previous work of our research team (44), in which the superior MPFC was positively associated with higher scores in the cognitive domain and negatively associated with higher scores in the affective domain.

In conclusion, this study provided some insights into the configuration of two key social networks across different brain states (resting vs. social task toward a close significant other). Taken together, our findings showed that both intra- and internetwork connectivity increased from resting to task, supporting the need for a higher integration between different social brain areas during the active processing of social information. On the other hand, the focus on the other's experience revealed limited connectivity within key SN nodes such as the AI and the dACC, emphasizing the connection between this network and self-referential processing.

## Limitations And Future Directions

The present work used two complementary functional connectivity methods to describe the relationship between the nodes of the DMN and the SN. FC methods are based on the correlations between the brain region's BOLD signal fluctuations over time, and despite its utility and extensive use in the literature, they can be complemented by other approaches. One of those complementary methods is dynamic functional connectivity, which, contrarily to FC that is based on the assumption of stationarity, addresses the temporal component (fluctuations) of spontaneous BOLD signals (60, 83). On the other hand, despite the ability of traditional FC methods to detect consistent spatiotemporal relationships between different brain regions, they do not assess the direct influence that one brain region exerts over another. This can be done through effective connectivity analysis (84) that, as showed in our previous work (67), considers how the information flows through the brain regions of a given network as well as between networks (85). For example, the knowledge of the information flow between socio-affective and socio-cognitive networks will clarify if these networks are hierarchically related, with the ability to abstract mental state attributions being dependent on the ability to simulate the other state.

The relative homogeneity of our sample in terms of age, relationship duration, and marital functioning may also be seen as a possible limitation of the present work, limiting the generalization of our findings to similar samples of relatively young and healthy couples. This may be important considering that variables such as the duration of the participants' relationship and the associated level of interpersonal closeness may modulate the overlap between self and other neural representations. For example, Cheng et al. (13) found that the closer the participants were with their partner, the greater the deactivation in the right TPJ and the lesser the self-other overlap. Likewise, López-Solà et al. (29) found that greater interpersonal closeness between partners predicted greater vicarious pain responses. Thus, future studies should measure [using questionnaires such as the Inclusion of the other in the self scale (86)] or experimentally manipulate relationship factors that may modulate the cognitive and affective routes of SC toward close others.

Finally, and although this study may have implications for couples' research, it would be interesting to examine the existence of similar connectivity patterns in other human dyads, such as parent–child or therapist–patient exchanges.

## Data Availability Statement

The raw data supporting the conclusions of this article will be made available by the authors, without undue reservation.

## Ethics Statement

The studies involving human participants were reviewed and approved by Institutional Review Board of University of Minho. The patients/participants provided their written informed consent to participate in this study. The study complied with the principles expressed in the Declaration of Helsinki (with the amendment of Tokyo 1975, Venice 1983, Hong Kong 1989, Somerset West 1996, Edinburgh 2000).

## Author Contributions

JFC, JMS, and AS designed the study concept and design. JFC and PO-S collected data for the experiments. CRC performed the data analysis and interpretation under the supervision of JFC and JMS. JFC, CRC, and JMS wrote the manuscript. All authors reviewed and approved the final draft.

## Funding

This study was funded by BIAL Foundation (Grant No: 87/12) and by POCI-01-0145-FEDER-028682, PTDC/PSI-GER/32152/2017). This study was conducted at the Psychology Research Centre (PSI/01662), School of Psychology, University of Minho, and supported by the Portuguese Foundation for Science and Technology (FCT) through the Portuguese State Budget (Ref.: UIDB/PSI/01662/2020) and by the Portuguese Ministry of Education and Science through national funds and co-financed by FEDER through COMPETE2020 under the PT2020 Partnership Agreement (POCI-01-0145-FEDER-007653). AS was supported by FCT (POCI-01-0145-FEDER-028682, PTDC/PSI-GER/32152/2017). PO-S was supported by the BIAL Foundation (Grant No: 217/16) and by the FCT (Ref.: UIDB/04872/2020).

## Conflict of Interest

The authors declare that the research was conducted in the absence of any commercial or financial relationships that could be construed as a potential conflict of interest.

## Publisher's Note

All claims expressed in this article are solely those of the authors and do not necessarily represent those of their affiliated organizations, or those of the publisher, the editors and the reviewers. Any product that may be evaluated in this article, or claim that may be made by its manufacturer, is not guaranteed or endorsed by the publisher.

## References

[1] Lieberman MD Straccia MA Meyer ML Du M Tan KM. Social, self, (situational), and affective processes in medial prefrontal cortex (mPFC): causal, multivariate, and reverse inference evidence. Neurosci Biobehav Rev. (2019) 99:311–28. 10.1016/j.neubiorev.2018.12.021 30610911

[2] Schurz M Radua J Tholen MG Maliske L Margulies DS Mars RB. Toward a hierarchical model of social cognition: a neuroimaging meta-analysis and integrative review of empathy and theory of mind. Psychol Bull. (2020) 147:293–327. 10.1037/bul0000303 33151703

[3] Van Overwalle F. Social cognition and the brain: a meta- analysis. Hum Brain Mapp. (2009) 30:829–58. 10.1002/hbm.20547 18381770 PMC6870808

[4] Cohen S Schulz MS Weiss E Waldinger RJ. Eye of the beholder: the individual and dyadic contributions of empathic accuracy and perceived empathic effort to relationship satisfaction. J Family Psychol. (2012) 26:236–45. 10.1037/a0027488 22369462

[5] Péloquin K Lafontaine MF. Measuring empathy in couples: validity and reliability of the interpersonal reactivity index for couples. J Pers Assess. (2010) 92:146–57. 10.1080/00223890903510399 20155564

[6] Waldinger RJ Hauser ST Schulz MS Allen JP Crowell JA. Reading others emotions: the role of intuitive judgments in predicting marital satisfaction, quality, and stability. J Family Psychol. (2004) 18:58–71. 10.1037/0893-3200.18.1.58 14992610 PMC1770839

[7] Kanske P Böckler A Trautwein F-M Singer T. Dissecting the social brain: introducing the EmpaToM to reveal distinct neural networks and brain-behavior relations for empathy and theory of mind. Neuroimage. (2015) 122:6–19. 10.1016/j.neuroimage.2015.07.082 26254589

[8] Kanske P Böckler A Trautwein F-M Parianen Lesemann FH Singer T. Are strong empathizers better mentalizers? Evidence for independence and interaction between the routes of social cognition. Soc Cogn Affect Neurosci. (2016) 11:1383–92. 10.1093/scan/nsw052 27129794 PMC5015801

[9] de Vignemont F Singer T. The empathic brain: how, when and why? Trends Cogn Sci. (2006) 10:435–41. 10.1016/j.tics.2006.08.008 16949331

[10] Walter H. Social cognitive neuroscience of empathy: concepts, circuits, and genes. Emot Rev. (2012) 4:9–17. 10.1177/1754073911421379

[11] Frith C Frith U. Theory of mind. Curr Biol. (2005) 15:R644–5. 10.1016/j.cub.2005.08.041 16139190

[12] Schurz M Radua J Aichhorn M Richlan F Perner J. Fractionating theory of mind: a meta-analysis of functional brain imaging studies. Neurosci Biobehav Rev. (2014) 42:9–34. 10.1016/j.neubiorev.2014.01.009 24486722

[13] Cheng Y Chen C Lin CP Chou KH Decety J. Love hurts: an fMRI study. Neuroimage. (2010) 51:923–9. 10.1016/j.neuroimage.2010.02.047 20188182

[14] Zaki J Weber J Ochsner K. Task-dependent neural bases of perceiving emotionally expressive targets. Front Hum Neurosci. (2012) 6:228. 10.3389/fnhum.2012.00228 22876229 PMC3410370

[15] Alcalá-López D Smallwood J Jefferies E Van Overwalle F Vogeley K Mars RB. Computing the social brain connectome across systems and states. Cereb Cortex. (2018) 28:2207–32. 10.1093/cercor/bhx121 28521007

[16] Valk SL Bernhardt BC Bockler A Trautwein F-M Kanske P. Socio-cognitive phenotypes differentially modulate large-scale structural covariance networks. Cereb Cortex. (2017) 27:1358–68. 10.1093/cercor/bhv319 26733538

[17] Alcalá-López D Vogeley K Binkofski F Bzdok D. Building blocks of social cognition: mirror, mentalize, share? Cortex. (2019) 118:4–18. 10.1016/j.cortex.2018.05.006 29903609

[18] Bzdok D Schilbach L Vogeley K Scheider K Laird AR Langner R. Parsing the neural correlates of moral cognition: ALE meta-analysis on morality, theory of mind, and empathy. Brain Struct Funct. (2012) 217:783–96. 10.1007/s00429-012-0380-y 22270812 PMC3445793

[19] Fan Y Duncan NW de Greck M Northoff G. Is there a core neural network in empathy? An fMRI based quantitative meta-analysis. Neurosci Biobehav Rev. (2011) 35:903–11. 10.1016/j.neubiorev.2010.10.009 20974173

[20] de Waal F Preston S. Mammalian empathy: behavioural manifestations and neural basis. Nat Rev Neurosci. (2017) 18:498–509. 10.1038/nrn.2017.72 28655877

[21] Chiong W Wilson SM D'Esposito M Kayser AS Grossman SN Poorzand P Seeley WW. The salience network causally influences default mode network activity during moral reasoning. Brain J. Neurol. (2013) 136:1929–41. 10.1093/brain/awt066 23576128 PMC3673466

[22] Menon V Uddin LQ. Saliency, switching, attention and control: a network model of insula function. Brain Struct Funct. (2010) 214:655–67. 10.1007/s00429-010-0262-0 20512370 PMC2899886

[23] Seeley WW Menon V Schatzberg AF Keller J Glover GH Kenna H. Dissociable intrinsic connectivity networks for salience processing and executive control. J Neurosci. (2007) 27:2349–56. 10.1523/JNEUROSCI.5587-06.2007 17329432 PMC2680293

[24] Menon V. Salience network. Brain Mapp. (2015) 2:597–611. 10.1016/B978-0-12-397025-1.00052-X

[25] Sevinc G Gurvit R Spreng RN. Salience network engagement with the detection of morally laden information. Soc Cogn Aff Neurosci. (2017) 12:1118–27. 10.1093/scan/nsx035 28338944 PMC5490682

[26] Fallon N Roberts C Stancak A. Shared and distinct functional networks for empathy and pain processing: a systematic review and meta-analysis of fMRI studies. Soc Cogn Aff Neurosci. (2020) 15:709–23. 10.1093/scan/nsaa090 32608498 PMC7511882

[27] Gu X Liu X Guise KG Naidich TP Hof PR Fan J. Functional dissociation of the frontoinsular and anterior cingulate cortices in empathy for pain. J Neurosci. (2010) 30:3739–44. 10.1523/JNEUROSCI.4844-09.2010 20220007 PMC2845539

[28] Timmers I Park AL Fischer MD Kronman CA Heathcote LC Hernandez JM. Is empathy for pain unique in its neural correlates? A meta-analysis of neuroimaging studies of empathy. Front Behav Neurosci. (2018) 12:289. 10.3389/fnbeh.2018.00289 30542272 PMC6277791

[29] López-Solà M Koban L Krishnan A Tor Wager TD. When pain really matters: a vicarious-pain brain marker tracks empathy for pain in the romantic partner. Neuropsychologia. (2020) 145:106427. 10.1016/j.neuropsychologia.2017.07.012 28712948

[30] Nomi JS Farrant K Damaraju E Rachakonda S Calhoun VD Uddin LQ. Dynamic functional network connectivity reveals unique and overlapping profiles of insula subdivisions. Hum Brain Mapp. (2016) 37:1770–87. 10.1002/hbm.23135 26880689 PMC4837017

[31] Craig ADB. The sentient self. Brain Struct Funct. (2010) 214:563–77. 10.1007/s00429-010-0248-y 20512381

[32] Schilbach L Bzdok D Timmermans B Fox PT Laird AR Vogeley K. Introspective minds: using ALE meta-analyses to study commonalities in the neural correlates of emotional processing, social and unconstrained cognition. PLoS ONE. (2012) 7:e30920. 10.1371/journal.pone.0030920 22319593 PMC3272038

[33] Di X Biswal BB. Identifying the default mode network structure using dynamic causal modeling on resting-state functional magnetic resonance imaging. Neuroimage. (2014) 86:53–9. 10.1016/j.neuroimage.2013.07.071 23927904 PMC3947265

[34] Greicius MD Krasnow B Reiss AL Menon V. Functional connectivity in the resting brain: a network analysis of the default mode hypothesis. Proc Natl Acad Sci USA. (2003) 100:253–8. 10.1073/pnas.0135058100 12506194 PMC140943

[35] Raichle ME. The brain's default mode network. Annu Rev Neurosci. (2015) 38:433–47. 10.1146/annurev-neuro-071013-014030 25938726

[36] Raichle ME MacLeod AM Snyder AZ Powers WJ Gusnard DA Shulman GL. A default mode of brain function. Proc Natl Acad Sci USA. (2001) 98:676–82. 10.1073/pnas.98.2.676 11209064 PMC14647

[37] Buckner RL. The serendipitous discovery of the brain's default network. Neuroimage. (2012) 62:1137–45. 10.1016/j.neuroimage.2011.10.035 22037421

[38] Amft M Bzdok D Laird AR Fox PT Schilbach L Eickhoff S R. Definition and characterization of an extended social-affective default network. Brain Struct Funct. (2015) 220:1031–49. 10.1007/s00429-013-0698-0 24399179 PMC4087104

[39] Li W Mai X Liu C. The default mode network and social understanding of others: what do brain connectivity studies tell us. Front Hum Neurosci. (2014) 8:74. 10.3389/fnhum.2014.00074 24605094 PMC3932552

[40] Spreng RN Andrews-Hanna JR. The default network and social cognition. Brain Mapp. (2015) 3:165–9. 10.1016/B978-0-12-397025-1.00173-1

[41] Mars RB Neubert FX Noonan MP Sallet J Toni I Rushworth MF. On the relationship between the “default mode network” and the “social brain”. Front Hum Neurosci. (2012) 6:189. 10.3389/fnhum.2012.00189 22737119 PMC3380415

[42] Sampaio A Soares JM Coutinho J Sousa N Gonçalves OF. The big five default brain: functional evidence. Brain Struct Funct. (2014) 219:1913–22. 10.1007/s00429-013-0610-y 23881294

[43] Coutinho JF Sampaio A Ferreira M Soares JM Gonçalves OF. Brain correlates of pro-social personality traits: a voxel-based morphometry study. Brain Imaging Behav. (2013) 7:293–9. 10.1007/s11682-013-9227-2 23512407

[44] Oliveira-Silva P Maia L Coutinho J Frank B Soares JM Sampaio A. Empathy by default: Correlates in the brain at rest. Psicothema. (2018) 30:97–103. 10.7334/psicothema2016.366 29363477

[45] Esménio S Soares JM Oliveira-Silva P Zeidman P Razi A Goncalves OF. Using resting-state DMN effective connectivity to characterize the neurofunctional architecture of empathy. Sci Rep. (2019) 9:2603. 10.1038/s41598-019-38801-6 30796260 PMC6385316

[46] Meyer ML. Social by default: characterizing the social functions of the resting brain. Curr Dir Psychol Sci. (2019) 28:380–6. 10.1177/0963721419857759

[47] Northoff G Duncan NW Hayes DJ. The brain and its resting state activity — experimental and methodological implications. Progress Neurobiol. (2010) 92:593–600. 10.1016/j.pneurobio.2010.09.002 20888388

[48] Schilbach L Eickhoff SB Rotarska-Jagiela A Fink GR Vogeley K. Minds at rest? Social cognition as the default mode of cognizing and its putative relationship to the “default system” of the brain. Consciousness Cogn. (2008) 17:457–67. 10.1016/j.concog.2008.03.013 18434197

[49] Meyer ML Lieberman MD. Why people are always thinking about themselves: medial prefrontal cortex activity during rest primes self-referential processing. J Cogn Neurosci. (2018) 30:714–21. 10.1162/jocn_a_01232 29308983

[50] Esménio S Soares JM Oliveira-Silva P Gonçalves Ó. F Decety J. Brain circuits involved in understanding our own and other's internal states in the context of romantic relationships. Soc Neurosci. (2019) 14:729–38. 10.1080/17470919.2019.1586758 30806571

[51] Huang Z Obara N DavisIV HH Pokorny J Northoff G. The temporal structure of resting-state brain activity in the medial prefrontal cortex predicts self-consciousness. Neuropsychologia. (2016) 82:161–70. 10.1016/j.neuropsychologia.2016.01.025 26805557

[52] Krienen FM Tu -C P Buckner RL. Clan mentality: evidence that the medial prefrontal cortex responds to close others. J Neurosci. (2010) 30:13906–15. 10.1523/JNEUROSCI.2180-10.2010 20943931 PMC2989424

[53] Courtney AL Meyer ML. Self-other representation in the social brain reflects social connection. J Neurosci. (2020) 40:5616–27. 10.1523/JNEUROSCI.2826-19.2020 32541067 PMC7363469

[54] Preckel K Kanske P Singer T. On the interaction of social affect and cognition: empathy, compassion and theory of mind. Curr Opin Behav Sci. (2018) 19:1–6. 10.1016/j.cobeha.2017.07.01033421860

[55] Schurz M Maliske L Kanske P. Cross-network interactions in social cognition: a review of findings on task related brain activation and connectivity. Cortex. (2021) 130:142–57. 10.1016/j.cortex.2020.05.006 32653744

[56] Meyer ML Masten CL Ma Y Wang C Shi Z Eisenberger NI. Empathy for the social suffering of friends and strangers recruits distinct patterns of brain activation. Soc Cogn Affect Neurosci. (2013) 8:446–54. 10.1093/scan/nss019 22355182 PMC3624958

[57] Eddy CM. The junction between self and other? temporo-parietal dysfunction in neuropsychiatry. Neuropsychologia. (2016) 89:465–77. 10.1016/j.neuropsychologia.2016.07.030 27457686

[58] Santiesteban I Banissy MJ Catmur C Bird G. Enhancing social ability by stimulating right temporoparietal junction. Curr Biol. (2012) 22:2274–7. 10.1016/j.cub.2012.10.018 23122848

[59] Von dem Hagen EAH Stoyanova RS Baron-Cohen S Calder AJ. Reduced functional connectivity within and between “social” resting state networks in autism spectrum conditions. Soc Cogn Affect Neurosci. (2013) 8:694–701. 10.1093/scan/nss053 22563003 PMC3739917

[60] Soares JM Magalhães R Moreira PS Sousa A Ganz E Sampaio A. A hitchhiker's guide to functional magnetic resonance imaging. Front Neurosci. (2016) 10:515. 10.3389/fnins.2016.00515 27891073 PMC5102908

[61] Nieto-Castanon A. Handbook of Functional Connectivity Magnetic Resonance Imaging Methods in CONN (2020). Available online at: https://www.researchgate.net/publication/339460691_Handbook_of_functional_connectivity_Magnetic_Resonance_Imaging_methods_in_CONN.

[62] Murray RJ Debbané M Fox PT Bzdok D Eickhoff SB. Functional connectivity mapping of regions associated with self- and other-processing. Hum Brain Mapp. (2015) 36:1304–24. 10.1002/hbm.22703 25482016 PMC4791034

[63] Coutinho J Beiramar A Silva C Lema A Lima V Grace R. Evidências de validade da versão portuguesa do índice de reatividade interpessoal para casais. Avaliação Psicol. (2015) 14:309–17. 10.15689/ap.2015.1403.02

[64] Davis MH. A multidimensional approach to individual differences in empathy. JSAS Catalog Select Documents Psychol. (1980) 10:85.

[65] Coutinho J Oliveira-Silva P Mesquita AR Barbosa M Perrone-Mcgovern KM Gonçalves OF. Psychophysiological reactivity in couples during a marital interaction task. Appl Psychophysiol Biofeedback. (2017) 42:335–46. 10.1007/s10484-017-9380-2 28866813

[66] Coutinho J Oliveira-Silva P Fernandes E Gonçalves OF Correia D Perrone Mc-Govern K. Psychophysiological synchrony during verbal interaction in romantic relationships. Fam Process. (2018) 58:716–33. 10.1111/famp.12371 29888517

[67] Esménio S Soares JM Oliveira-Silva P Gonçalves Ó F Friston K. Changes in the effective connectivity of the social brain when making inferences about close others vs. the self. Front Hum Neurosci. (2020) 14:151. 10.3389/fnhum.2020.00151 32410974 PMC7202326

[68] Chao-Gan Y Yu-Feng Z. DPARSF: a MATLAB toolbox for “pipeline” data analysis of resting-state fMRI. Front Syst Neurosci. (2010) 4:13. 10.3389/fnsys.2010.00013 20577591 PMC2889691

[69] Ashburner J. A fast diffeomorphic image registration algorithm. Neuroimage. (2007) 38:95–13. 10.1016/j.neuroimage.2007.07.007 17761438

[70] Beckmann C DeLuca M Devlin JT Smith SM. Investigations into resting-state connectivity using independent component analysis. Philos Trans R SocB. (2005) 360:1001–13. 10.1098/rstb.2005.1634 16087444 PMC1854918

[71] Calhoun VD Adali T Pearlson GD Pekar JSpatial J. and temporal independent component analysis of functional MRI data containing a pair of task-related waveforms. Hum Brain Mapp. (2001) 13:43–53. 10.1002/hbm.1024 11284046 PMC6871956

[72] Bell AJ Sejnowski JT. An information-maximization approach to blind separation and blind deconvolution. Neural Comput. (1995) 7:1129–59. 10.1162/neco.1995.7.6.1129 7584893

[73] Himberg J Hyvärinen A Esposito F. Validating the independent components of neuroimaging time series via clustering and visualization. Neuroimage. (2004) 22:1214–22. 10.1016/j.neuroimage.2004.03.027 15219593

[74] Tzourio-Mazoyer N Landeau B Papathanassiou D Crivello F Etard O Delcroix N. Automated anatomical labeling of activations in SPM using a macroscopic anatomical parcellation of the MNI MRI single-subject brain. Neuroimage. (2002) 15:273–89. 10.1006/nimg.2001.0978 11771995

[75] Whitfield-Gabrieli S Nieto-Castanon A. Conn: a functional connectivity toolbox for correlated and anticorrelated brain networks. Brain Connect. (2012) 2:125–41. 10.1089/brain.2012.0073 22642651

[76] Wang Y Metoki A Xia Y Zang Y He Y Olson IR. A large-scale structural and functional connectome of social mentalizing. Neuroimage. (2021) 236:118115. 10.1016/j.neuroimage.2021.118115 33933599 PMC12317760

[77] Aminoff EM Kveraga K Bar M. The role of the parahippocampal cortex in cognition. Trends Cogn Sci. (2013) 17:379–90. 10.1016/j.tics.2013.06.009 23850264 PMC3786097

[78] Laurita AC Hazan C Spreng RN. Dissociable patterns of brain activity for mentalizing about known others: a role for attachment. Soc Cogn Affect Neurosci. (2017) 12:1072–82. 10.1093/scan/nsx040 28407150 PMC5490684

[79] Shine JM Bissett PG Bell PT Koyejo O Balsters JH Gorgolewski KJ. The dynamics of functional brain networks: integrated network states during cognitive task performance. Neuron. (2016) 92:544–54. 10.1016/j.neuron.2016.09.018 27693256 PMC5073034

[80] Shine JM Poldrack RA. Principles of dynamic network reconfiguration across diverse brain states. Neuroimage. (2018) 180:396–405. 10.1016/j.neuroimage.2017.08.010 28782684

[81] Sowden S Catmur C. The role of the right temporoparietal junction in the control of imitation. Cereb Cortex. (2015) 25:1107–1113. 10.1093/cercor/bht306 24177989 PMC4380005

[82] Qin P Wang M Northoff G. Linking bodily, environmental and mental states in the self—a three-level model based on a meta-analysis. Neurosci Biobehav Rev. (2020) 115:77–95. 10.1016/j.neubiorev.2020.05.004 32492474

[83] Cabral J Hugues E Sporns O Deco G. Role of local network oscillations in resting-state functional connectivity. Neuroimage. (2011) 57:130–9. 10.1016/j.neuroimage.2011.04.010 21511044

[84] Friston KJ. Functional and effective connectivity in neuroimaging: a synthesis. Hum Brain Mapp. (1994) 2:56–78. 10.1002/hbm.46002010725855820

[85] Friston KJ. Functional and effective connectivity: a review. Brain Connecivit. (2011) 1:13–36. 10.1089/brain.2011.0008 22432952

[86] Aron A Aron EN Smollan D. Inclusion of other in the self scale and the structure of interpersonal closeness. J Pers Soc Psychol. (1992) 63:16. 10.1037/0022-3514.63.4.596

